# Acoustic Reconstruction for Photothermal Imaging

**DOI:** 10.3390/bioengineering5030070

**Published:** 2018-08-29

**Authors:** Peter Burgholzer, Gregor Stockner, Guenther Mayr

**Affiliations:** 1 Research Center for Non Destructive Testing (RECENDT), 4040 Linz, Austria; 2 Josef Ressel Centre for Thermal NDE of Composites, University of Applied Sciences Upper Austria, 4600 Wels, Austria; Gregor.Stockner@fh-wels.at (G.S.); guenther.mayr@fh-wels.at (G.M.)

**Keywords:** infrared thermography, acoustic reconstruction, virtual wave concept, image reconstruction, inverse problem

## Abstract

Pulsed illumination of a sample, e.g., of a biological tissue, causes a sudden temperature increase of light absorbing structures, such as blood vessels, which results in an outgoing acoustic wave, as well as heat diffusion, of the absorbed energy. Both of the signals, pressure and temperature, can be measured at the sample surface and are used to reconstruct the initial temperature or pressure distribution, called photoacoustic or photothermal reconstruction respectively. We have demonstrated that both signals at the same surface pixel are connected by a temporal transformation. This allows for the calculation of a so-called acoustical virtual wave from the surface temperature evolution as measured by an infrared camera. The virtual wave is the solution of a wave equation and can be used to reconstruct the initial temperature distribution immediately after the excitation pulse. This virtual wave reconstruction method was used for the reconstruction of inclined steel rods in an epoxy sample, which were heated by a short pulse. The reconstructed experimental images show clearly the degradation of the spatial resolution with increasing depth, which is theoretically described by a depth-dependent thermographic point-spread-function.

## 1. Introduction

Photothermal imaging using an infrared camera for signal detection has a big advantage compared to ultrasonic imaging using acoustic transducers: it does not need any coupling media and parallel detection of many camera pixels is possible. The main disadvantage of pulsed thermography is the degrading spatial resolution with increasing depth. This is the reason why often only one-dimensional thermographic reconstruction is performed, which can be applied for layered structures where the lateral extension is big compared to their depth. For other structures, using a one-dimensional reconstruction gives additional artifacts as the heat propagates not only perpendicular to the surface but also in the lateral direction. Solving directly the inverse three-dimensional heat diffusion equation is rather ambitious, even for simple geometries of the embedded structure, e.g., rectangular vertical cracks. 

In this work, it is shown that image reconstruction methods from ultrasonic imaging can be employed for thermographic signals. Before using these imaging methods, a virtual signal is calculated by applying a local transformation to the temperature evolution measured on a sample surface. The introduced transformation describes all of the irreversibility of the heat diffusion process and can be used for every sample shape in two and three dimensions. The feasibility of this approach was demonstrated through experiments: Steel rods embedded in an epoxy resin were imaged. 

In acoustical or optical imaging, this blurring of structures is usually modeled by convolution with a point-spread-function. We have introduced such a point-spread-function for thermographic imaging, which shows that the lateral resolution is approximately twice the axial resolution. The resolution for pulsed thermography is proportional to the depth of the point source divided by the natural logarithm of the signal-to-noise ratio.

In photoacoustic (also known as thermoacoustic or optoacoustic) or photothermal imaging, usually a short pulse of electromagnetic radiation, such as a laser or a microwave pulse, illuminates a sample. The electromagnetic radiation is absorbed by various chromophores in different regions of the sample. The resultant rapid heating and thermal expansion produce an acoustic wave travelling to the sample surface, with sound velocity *c*, where it is measured as a pressure wave. In parallel, but usually much slower, the heat absorbed from the pulse diffuses into the sample and the resultant temperature evolution can be measured on the sample surface using an infrared camera. Both of the measured surface signals, pressure and temperature, can be used to reconstruct the initial temperature or pressure distribution, called photoacoustic or photothermal reconstruction respectively. We have demonstrated that both of the signals for the same sample location, especially for a surface point where the temporal temperature evolution was observed by a single pixel of an infrared camera, are connected by a temporal transformation. This transformation allows for the calculation, from the measured surface temperature evolution, of a so-called acoustical virtual wave, which is the solution of the wave equation and can be used to reconstruct the initial temperature distribution immediately after the excitation pulse [1]. 

The heat diffusion is mathematically described by the diffusion equation:

(1) $$(\nabla^{2}-\frac{1}{\alpha }\frac{\partial }{\partial t})T(r,t)=-\frac{1}{\alpha }T_{0}\left(r\right)\delta \left(t\right),$$

with temperature distribution *T*(***r****,t*) in space and time, ∇^2^ the Laplace-operator (second derivative in space), and the thermal diffusivity *α* which is assumed to be homogeneous in the sample. The source-term on the right side of Equation (1) ensures that *T*_0_(***r***) gives the initial temperature distribution immediately after the short pulse. The temporal excitation is described by the Dirac-Delta-function *δ*(*t*).

The propagation of the acoustic wave with pressure *p*(***r****,t*) is described by the wave equation [2,3]:

(2) $$(\nabla^{2}-\frac{1}{c^{2}}\frac{\partial^{2}}{\partial t^{2}})p(r,t)=-\frac{1}{c^{2}}\frac{\partial }{\partial t}p_{0}\left(r\right)\delta \left(t\right)$$

with the sound velocity *c* and the generated acoustic pressure *p*_0_(***r***) immediately after the excitation pulse through thermoelastic expansion.

$$p(r,t=0)=p_{0}(r)=c^{2}\rho \beta T_{0}(r),\;\mathrm{for}\;t=0,$$

β is the volumetric thermal expansion and ρ is the density of the sample. 

The same relation is extended to all times to define a virtual wave *T*_virt_(***r****,t*):

(3) $$T_{virt}\left(r,t\right)=\frac{p\left(r,t\right)}{\beta c^{2}\rho },$$

This virtual wave, therefore, obeys the wave equation:

(4) $$(\nabla^{2}-\frac{1}{c^{2}}\frac{\partial^{2}}{\partial t^{2}})T_{virt}(r,t)=-\frac{1}{c^{2}}\frac{\partial }{\partial t}T_{0}\left(r\right)\delta \left(t\right),$$

and we have shown that for every sample location ***r*** (local transformation) there is a relation between the measured temperature signal and this defined virtual wave *T*_virt_(***r****,t*) [1]:

(5) $$T(r,t)=\int_{-\infty }^{\infty }T_{virt}(r,t^{\prime })K\left(t,t^{\prime }\right)dt^{\prime },$$

$$\mathrm{with}\;K(t,t^{\prime })=\frac{c}{\sqrt{\pi \alpha t}}exp\left(-\frac{c^{2}t^{\prime^{2}}}{4at}\right),\;\mathrm{for}\;t>0$$

This local transformation is valid for every dimension of space and combines acoustic and thermal imaging, as kernel *K* contains sound velocity *c* as well as thermal diffusivity *α*. One of the main differences between heat diffusion and acoustic wave propagation is that the diffusion is an irreversible process producing entropy, whereas the acoustic wave, Equation (2), can be inverted in time—the equation has only a second derivative in time—which describes a reversible process without entropy production. Equation (5) contains all the irreversibility.

In the next section, it is shown how this entropy production and the corresponding information loss causes a principal loss in spatial resolution at higher depths. In the following sections, inclined steel rods embedded in an epoxy sample were heated by a short pulse. Simulation results, experimental data and reconstructed images are shown.

## 2. Methods and Materials

### 2.1. Entropy Production, Information Loss and Spatial Resolution

As already mentioned in the introduction, Equation (5) is a local transformation. For the same sample location ***r***, it is a transformation between the virtual wave *T*_virt_(***r****,t*), which is a reversible solution to the wave Equation (2), and the temperature *T*(***r****,t*), which is an irreversible solution of the diffusion Equation (1). Therefore, the calculation of *T*(***r****,t*) from *T*_virt_(***r****,t*) using Equation (5), which can be written for a discretized time as a matrix multiplication [1] discards information. The matrix for kernel *K* is not directly invertible, which is the mathematical consequence of the loss of information through entropy production during the heat diffusion process. Regularization methods such as the truncated singular value decomposition (T-SVD) method can be used to determine the (pseudo-) inverse matrix of the discrete kernel *K* [1]. The inverse problem, that the calculation of *T_virt_*(***r****,t*) from *T*(***r****,t*) is ill-posed, or the rank of the discretized kernel matrix is less than its size, because of the lost information. Poor discretization, low-quality infrared cameras or noisy amplifiers can increase the loss of information. However, even if all of these “technical” limitations can be neglected, the lost information equal to entropy production of heat diffusion cannot be recovered by any mathematical reconstruction algorithm and results in a loss of spatial resolution. 

Thermodynamic fluctuations are the reason for the entropy production, which reduces the available information about the subsurface structures in the measured surface data. They are extremely small for macroscopic samples but are highly amplified due to the ill-posed problem of image reconstruction. For macroscopic samples, the resolution limit depends only on the amplitude of these fluctuations. The thermodynamic fluctuations and the entropy production caused by heat diffusion are strongly connected by the fluctuation-dissipation theorem. They are the “two sides of the same coin” of heat diffusion. Therefore, the truncation index for the regularization of the inverse problem using the T-SVD method is closely connected to the fluctuations, expressed by the signal-to-noise ratio (*SNR*). If the singular value of the kernel *K* is ordered in a monotonically decreasing order, the last singular value which is not set to zero (with the index equal to the truncation index) divided by the first singular value should be equal to the *SNR* (up to rounding errors).

In optics, it has been known since 2007 that optical diffusion in a strongly scattering sample can be “inverted” by wave front shaping [4]. Coherent photons from a laser scatter in a deterministic way (at least for a certain correlation time), which is measured in a pointwise way. The resultant scattering matrix is used to change the light wave front, e.g., by a spatial light modulator in such a way that after the diffusive scattering the light is focused. For heat diffusion, phonons are not coherent and the propagation of phonons is highly uncorrelated in time. Therefore, a “phonon scattering matrix” cannot be measured and used to invert heat diffusion in time.

Non-equilibrium thermodynamics has made enormous progress the last decade. Heating light absorbing structures with a laser pulse and observing the temperature diffusion is definitely a process where the system state is far from equilibrium. One comprehensive letter about non-equilibrium thermodynamics, the second law and the connection between entropy production and information loss, was published in 2011 by Esposito and van den Broeck [5]. They showed that for two different non-equilibrium states evolving to the same equilibrium state the entropy production Δ_i_S during the evolution from one state to the other is equal to the information loss ΔI = k_B_ΔD, where k_B_ is the Boltzmann constant and ΔD is the difference of the Kullback-Leibler divergence D, also called relative entropy of these states. D is a measure of how “far” a certain state is away from equilibrium [6]. The entropy production for the macroscopic states with small fluctuations around equilibrium turns out to be, in a good approximation, equal to the dissipated energy ΔQ, which is the heat transported by heat diffusion divided by the mean temperature T_mean_, so Δ_i_S = ΔQ/T_mean_ = k_B_ΔD [7].

In Fourier-space, the information loss with increasing time can be described by a cut-off wavenumber, *k_cut_* [7]. Spatial Fourier transformation of the heat diffusion Equation (1) shows that after some time, *t*, the heat diffusion reduces each Fourier component by a factor of exp(−*k*^2^*αt)*. Therefore, after a long time only the component with a wavenumber *k* = 0 is different from zero, which shows that in the thermal equilibrium, the temperature is the same everywhere in the sample. The information content, which is the negative entropy, of each Fourier component is ΔS_k_ = 0.5k_B_ × *SNR*^2^ exp(−2*k*^2^*αt)*, with the signal-to-noise ratio *SNR*. Now the cut-off wavenumber, *k_cut_,* is defined in such a way that the information content in this Fourier component is so low that its distribution cannot be distinguished from the equilibrium distribution within a certain statistical error level (Chernoff-Stein Lemma [6]). This error level can be set such that for wave numbers higher than *k_cut_* the Fourier components are dampened below the noise level, which gives:

$$SNR\;\exp(-k_{cut}{}^{2}\alpha t)=1$$

This is a principle thermodynamic limit for how good the initial temperature distribution can be reconstructed after some time. No mathematical regularization method can reconstruct information which has been discarded by the heat diffusion process.

In thermography, usually a temperature distribution is not measured at a certain time and the original temperature distribution *T*_0_(***r***) has to be reconstructed; but at certain locations at the sample surface, the temperature evolution for a certain time was measured and from that, *T*_0_(***r***) was reconstructed. For this case, we have shown that the spatial resolution limit is proportional to the depth and inversely proportional to the signal to noise ratio [8].

### 2.2. Experimental Set Up

**Figure 1:**
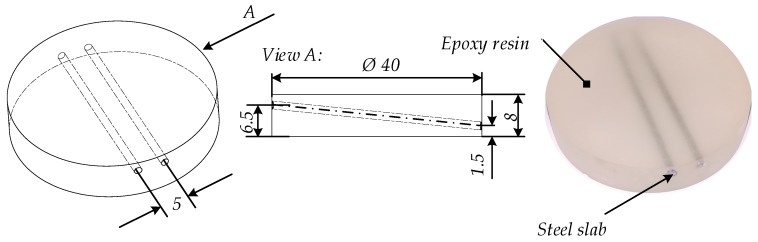
Principal sketch of the specimen. The specimen consists of steel rods that were embedded in epoxy resin. The cylindrical steel rods had a diameter of 1.5 mm.

For the experimental investigations, two cylindrical steel rods were embedded into epoxy resin, as shown in Figure 1. The embedded cylindrical steel rods had a diameter of 1.5 mm. The distance between the two steel rods along their cylindrical axis was 5 mm. The minimum distance between steel rods and the specimen surface was 0.75 mm in the *z*-direction. The embedding material had radii of 20 mm and a thickness of L = 8 mm. The sample was not excited optically by a laser pulse but by induction of eddy current. For other samples such as a graphite slab embedded into an optically scattering epoxy matrix, we have shown that laser and eddy current excitation produced similar results. Here, using eddy current, a more homogeneous heating on a larger volume was possible. We used the induction generator HÜTTINGER 2, 5/300 (TRUMPF Hüttinger, Freiburg, Germany) with a power of 3 kW at a frequency of 200 kHz. The heating time was *t_h_* = 2 s, applying the maximum power of the generator.

**Figure 2:**
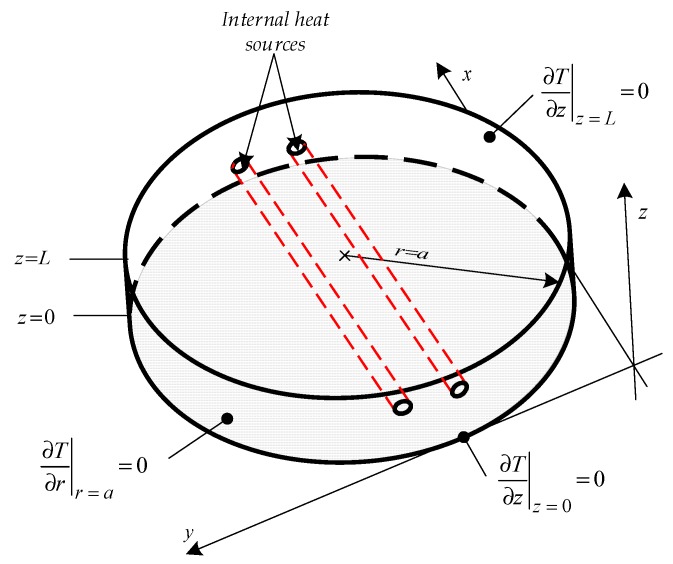
Boundaries that were applied to the experimental setup.

The specimen was positioned in the center of the inductor coils, which had a diameter of 80 mm. The specimen was thermally isolated at the lateral area and at the rear side to minimize heat losses due to convection and radiation. Hence it can be postulated that for each surface adiabatic boundary conditions apply, as illustrated in Figure 2. The temporal temperature distribution, at the front side of the specimen, was measured using an infrared camera that is sensitive in the spectral range of 3 to 5.1 mm. The infrared camera had a cooled indium antimonide InSb quantum detector with a focal plane array FPA of 1280 × 1024 pixels. The noise equivalent temperature difference (NETD) of the camera was 25 mK.

**Figure 3:**
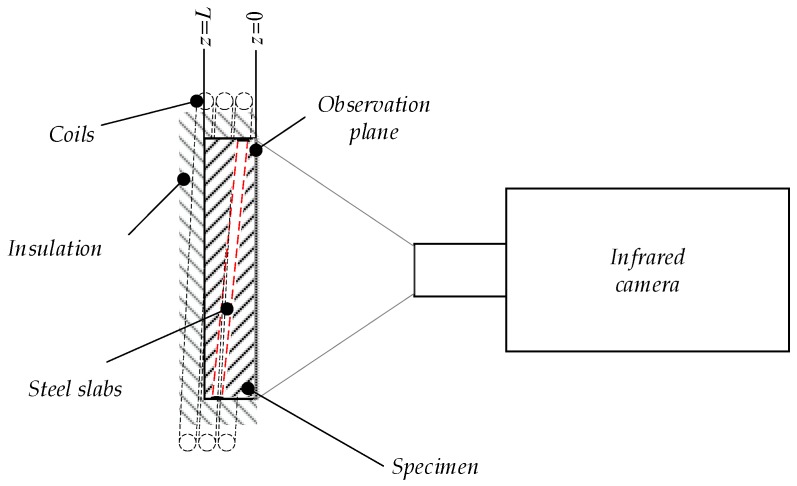
Principal sketch of the experimental set up to obtain experimental data for the validation of the acoustic reconstruction of infrared measurement data.

**Figure 4:**
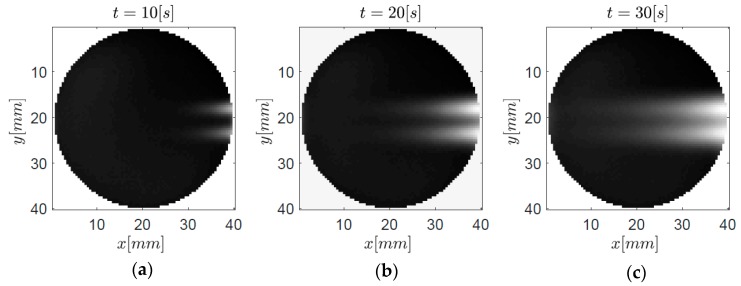
Thermograms at the front surface of the specimen at different time steps (**a**) 10 s, (**b**) 20 s, and (**c**) 30 s based on experimental data collected at the observation plane z = 0.

In Figure 3, the principal sketch of the measurement set up is depicted. The spatial resolution of the infrared camera was 167 µm and the measurement time was 200 s with a frame rate of 12.5 Hz. According to the applied discretization, the virtual speed of sound was *c* = 6.3 × 10^−3^ m/s. Due to the opacity of the epoxy resin in the medium infrared regime, the surface temperature of the specimen was measured and not the surface temperature of the steel rods. To increase the *SNR*, the mean value of nine adjacent pixels was taken, whereby the spatial resolution (*Δx*, *Δy*) was degenerated to 0.5 mm. The measured temperature distribution at the front surface of the sample is illustrated in Figure 4 for different cooling times *t* ((a) 10s, (b) 20s, and (c) 30s).

With increasing cooling time, it becomes very difficult to distinguish the embedded cylinders due to the decreasing resolution with increasing depth. Based on these temperature data, the virtual wave concept was applied to reconstruct the location and the initial temperature distribution *T_0_*(**r**) of the embedded cylindrical steel rods. Therefore, the virtual temperature field must be calculated from the measured temperature field and hence some kind of regularization must be introduced as stated above. To calculate the discrete kernel, the physical parameters in Table 1 were utilized. To compute the pseudo-inverse of the kernel, the T-SVD was used. 

## 3. Results

**Figure 5:**
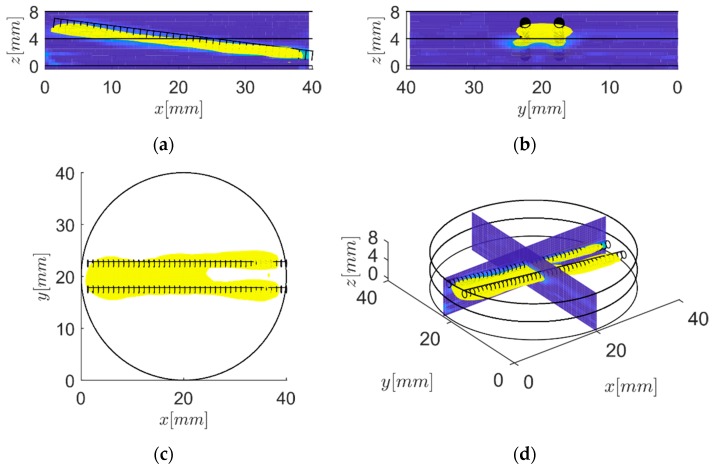
Visualization of the reconstructed cylindrical steel rods. The black circles indicate the target shape. The cross-section of the steel rods was elliptical. This is a consequence of the better resolution in depth compared to the lateral direction. (**a**) Front view. (**b**) Side view. (**c**) Top view. (**d**) 3D view.

Based on the virtual temperature field the internal volumetric heat sources can be reconstructed. Therefore, the Frequency Domain-Synthetic Aperture Focusing Technique (F-SAFT) algorithm is applied to the virtual temperature field [1]. Due to the F-SAFT, the initial temperature distribution *T_0_*(**r**) of the internal heat sources was reconstructed. The result is depicted in Figure 5. Herein, the black circles indicate the target shape and the yellow contours indicate the reconstruction. In Figure 5a, the reconstruction field at *y* = 22.5 mm, along the steel rod is shown. Herein, one can see that the depth of the reconstructed internal heat sources fits well. In Figure 5b, the reconstruction field at *x* = 20 mm, normal to the steel rods, is depicted. In Figure 5c a top view of the reconstruction field is illustrated. In the top view, it is directly visible that the internal heat sources cannot be separated in the range 0 ≤ *x* ≤ 25 mm.

The cylindrical cross-section of the steel rods, with respect to the reconstructed shape, appears elliptical due to the better resolution in depth direction in comparison with the lateral direction. Based on a thermal point spread function (PSF), it can be derived that the lateral resolution was a factor of 2.4 higher compared to the transversal resolution [8].

**Figure 6:**
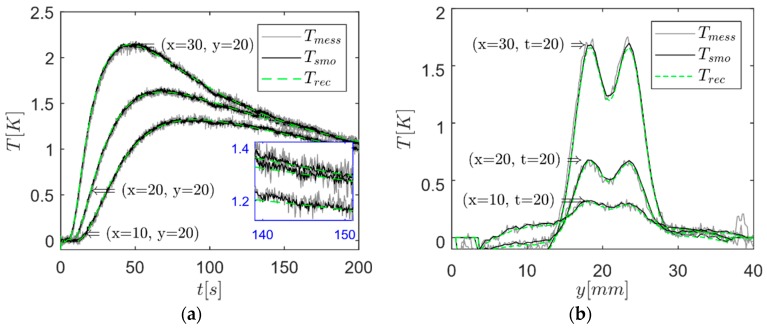
(**a**) Temporal temperature distributions at different positions *x* and *y* based on experimental data; (**b**) temperature field normal to the rods for *t* = 20 s at different *x* positions based on experimental data.

Nine neighbored pixels were averaged to reduce the noise level from 25 mK for each camera pixel to 8.33 mK. For the maximum of the measured temperature field of 3.12 K, this gives an *SNR* of 375. In Figure 6a, the temporal temperature with respect to the experimental data and reconstructed data is depicted for different positions, where *T*_mess_ is the measured temperature at (*x*,*y*), *T*_smo_ is the averaged measurement over nine neighbored pixels, and the reconstructed temperature field *T*_rec_ was calculated from the virtual wave using Equation (5). The zoom at the lower right position emphasizes the noise of the experimental temperature data. Herein, one can see the excellent filter effect of the T-SVD. Figure 6b shows the experimental and reconstructed temperature field normal to the steel rods for *t* = 20 s at different *x* positions.

## 4. Conclusions, Discussion, and Outlook

The virtual wave concept was used to reconstruct the initial temperature distribution inside an epoxy sample from the measured surface temperature evolution after heating with a short pulse. In the first step, from the measured temperature data, the virtual temperature data was reconstructed by inversion of Equation (5). As the second step, an ultrasound reconstruction method (F-SAFT) was used for reconstruction. The sample were two tilted parallel steel rods at a distance of 5 mm with increasing depth embedded in epoxy, which was heated for 2 s. The total measurement time of the surface temperature was 200 s. 

The reconstruction results in Figure 5 show that the spatial resolution decreases with increasing depth. At a depth deeper than approximately 4 mm, the two rods cannot be separated anymore, and the reconstruction shows just one rod. The cylindrical cross-section of the steel rods, with respect to the reconstructed shape, appears elliptical due to the better resolution in the depth direction compared to in the lateral direction (Figure 5b). 

To conclude, the presented virtual wave reconstruction method allows for the inversion of thermal diffusion up to a certain limit given by the entropy production. The degradation of resolution with increasing depth for thermographic imaging is a principle resolution limit, due to the heat diffusion producing entropy equal to the loss of information. This can be seen in the first step when the virtual wave was reconstructed from the actual temperature measurement data by the truncated singular-value decomposition method. The virtual wave signal became more blurred at later measurement times, corresponding to higher depths. The second step, the ultrasound reconstruction added no additional blurring to the reconstructions if the discretization in space and time was chosen properly.

The physical reason for entropy production and information loss is thermodynamic fluctuations as temperature is a statistical phenomenon. The fluctuations in macroscopic samples are usually so small that they can be neglected—but not for ill-posed inverse problems such as the thermographic reconstruction of internal temperature sources. Fluctuations and entropy production are closely related by the fluctuation-dissipation-theorem. A simple example of this fluctuation-dissipation-theorem between fluctuations in space (displacement) in Brownian motion and the diffusion constant has already been found by Einstein in 1905.

For future applications, photothermal imaging can be described as a convolution with a depth-dependent thermographic point-spread-function [8]. Together with structured illumination, e.g., by laser speckles inside a light-scattering medium the presented two-stage imaging algorithm (local transform to a virtual wave signal and then ultrasound reconstruction), allows for super-resolution photothermal imaging [8]. This circumvents the main disadvantage of photothermal imaging: the strong degrading spatial resolution with increasing depth.

## Figures and Tables

**Table 1:** Physical parameters of epoxy resin.

Parameter	Value	Unit
Thermal conductivity *k*	0.18	W/(m·K)
Specific heat capacity *c_p_*	1700	J/(kg·K)
Density *ρ*	1300	kg/(m^3^)
